# Determining Tyre Adhesion Characteristics Based on the Road Tests of Automobiles

**DOI:** 10.3390/s24237447

**Published:** 2024-11-22

**Authors:** Andrzej Reński, Mateusz Brukalski, Hubert Sar, Michał Abramowski, Piotr Fundowicz, Krzysztof Rokicki

**Affiliations:** Institute of Vehicles and Construction Machinery Engineering, Warsaw University of Technology, 84 Narbutta Str., 02-524 Warsaw, Poland; andrzej.renski@pw.edu.pl (A.R.); mateusz.brukalski@pw.edu.pl (M.B.); hubert.sar@pw.edu.pl (H.S.); piotr.fundowicz@pw.edu.pl (P.F.); krzysztof.rokicki@pw.edu.pl (K.R.)

**Keywords:** adhesion characteristics, longitudinal adhesion coefficient, longitudinal slip, CAN bus, active safety

## Abstract

The motion of automobiles significantly depends on the conditions of interaction between a tyre and a road surface. One of the most frequently used ways of presenting the conditions of cooperation between a tyre and a road surface is a characteristic showing a longitudinal adhesion coefficient as a function of a longitudinal slip of a tyre. One of the methods for determining tyre-to-road adhesion characteristics is to use a special trailer combined with a towing vehicle. This type of method is commonly used to determine adhesion characteristics for a braked wheel. This article presents a method for determining adhesion characteristics for a driven wheel based on the road tests of automobiles. For this purpose, vehicle wheel velocity signals from a vehicle CAN network and a vehicle velocity signal from a GPS receiver were used. The signals from the CAN network were recorded using a special measurement card and an application developed in LabVIEW environment. The application developed in LabVIEW also allowed for simultaneous recording of automobile velocity from the GPS receiver. In this paper, the courses of a wheel velocity, longitudinal acceleration of automobile, longitudinal slip of the front wheels in time domain, as well as the coefficient of tyre-to-road longitudinal adhesion as a function of the longitudinal slip of the wheel are presented.

## 1. Introduction

**Figure 1:**
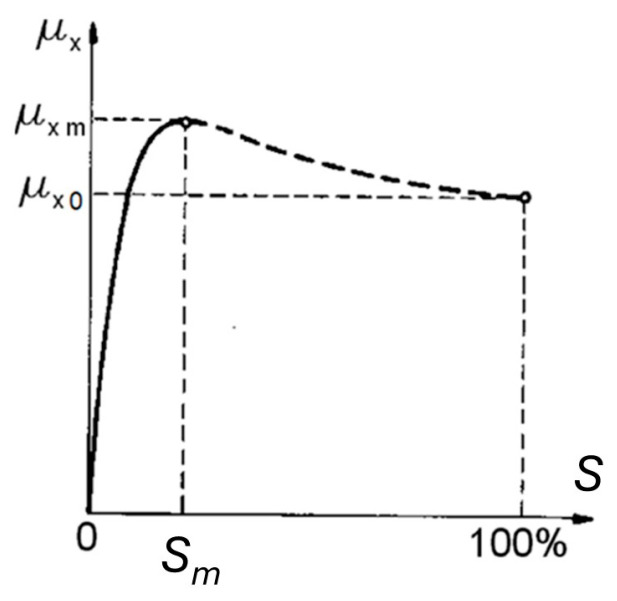
Dependence of the longitudinal adhesion coefficient *μ_x_* on slip *S*.

The interaction of automobile tyres with a road surface, and in particular their ability to transfer the tangential forces, is the factor that determines traffic safety. The conditions of cooperation between the tyre and the road surface when transmitting longitudinal forces (braking and acceleration) are graphically presented on a graph of the longitudinal adhesion coefficient as a function of slip *μ_x_*(*S*) (Figure 1). The longitudinal adhesion coefficient *μ_x_* is defined as the ratio of the longitudinal force between the road surface and the tyre *X* to the vertical reaction *Z*.

(1) $$\mu_{x}=\frac{X}{Z},$$

The slip is defined differently for drive and braking. The slip of the driven wheel:

(2) $$S=\frac{r_{d}\cdot \omega_{k}-v_{k}}{r_{d}\cdot \omega_{k}}100\%,$$

where

*v_k_*—velocity of the centre of the wheel, equal to a vehicle velocity;
*ω_k_*—angular velocity of a wheel;
*r_d_*—wheel dynamic radius.

And the slip of the braked wheel:

(3) $$S=\frac{v_{k}-r_{d}{\cdot \omega }_{k}}{v_{k}}100\%,$$

There are two characteristic points on the chart:

*μ_xm_*—the maximum value of the adhesion coefficient, called the peak adhesion coefficient, and the slip value at which it occurs is marked *S_m_*.
*μ_x_*_0_—adhesion coefficient at 100% slip, called the lockup adhesion coefficient.

From the point of view of automotive safety, it is important to be able to constantly monitor the adhesion of its wheels to the road surface. Modern automobiles are equipped with a number of systems whose task is to support the driver actions and correct his errors, which work by correcting the longitudinal force (driving or braking). The operation of these systems (ABS, EBD, ASR, ESP…) could be more effective if it was possible to provide them with information about the current value of the adhesion coefficient or the value of the longitudinal slip.

The aim of the research presented in this study was to verify whether it is possible to assess the adhesion of a vehicle’s wheels to the road surface based on the signals available in automobile or signals that are easy to measure without the need to install advanced equipment. Road measurements presented here were intended to record signals enabling the determination of the current value of the driven wheel adhesion coefficient and its slip.

When driving at constant velocity, limited by traffic regulations, the driving force balancing the rolling resistance force of the undriven wheels and the air drag force are relatively small, which means that, with high adhesion, the wheel slip is also low. Higher slip can be expected during dynamic acceleration, when the driving force is higher, and also when driving on a slippery road with lower adhesion. From a practical point of view, the detection of the increased slip of the driven wheels may be a signal indicating that the vehicle has entered a section of a road with reduced adhesion (layer of water, black ice, oil stain) and may be used to warn the driver about a potential hazard.

## 2. Methods for Determining Adhesion Characteristics

The course of adhesion characteristics is different for each pair: the tyre–road surface and may be different for the driven and braked wheel. The course of the graph for a specific pair is determined experimentally, and the method of carrying out the measurements also depends on the purpose for which the research is carried out. Thus, in road engineering, the value of lockup adhesion coefficient, i.e., adhesion coefficient with a locked wheel, determined on a surface covered with a layer of water, is important to assess anti-slip properties of the road surfaces [1]. In turn, in automotive technology, the value of the peak adhesion coefficient, also measured on wet surfaces, is used to evaluate the tyre [2]. Both in road construction and automotive technology, special measurement systems [3] in the form of trailers (for example, SRT-4—Skid Resistance Tester -4 [4]) or specially adapted vehicles [5] are most often used to determine the adhesion of the wheels to the road surface. An example of the use of a trailer in testing the tyre-to-road adhesion is the article [6]. In this case, the coefficient of lateral adhesion of the tyre to the road surface in relation to the given side slip angle of a wheel as well as various vertical loads on a wheel were also tested.

The article [7] presents a method for determining the tyre-to-road adhesion coefficient based on direct measurements of the forces at the contact between the tyre and the road surface, using special measuring wheels. Moreover, modified Pacejka’s Magic Formula equation is presented to include the effects of the contact such as road composition and its state, tyre type, vehicle speed, and tyre–road surface slip.

The work [8] presents a method for estimating the adhesion coefficient of a tyre to a road surface and its application in controlling the suspension of an automobile. Presented considerations use a three-axis acceleration sensor located inside a pneumatic wheel. Furthermore, the authors of the work [8] explain the role of the brush model describing the dependence between the tyre force and the slip including two parameters—tyre stiffness and tyre-to-road surface friction coefficient.

The article [9] presents a method for estimating longitudinal tangential forces at the contact between a tyre and a road surface, sensitive to the changes in a type of road surface.

In order to optimize the operation of an ABS system in various adhesion conditions, the article [10] presents research on the adhesion of a tyre to a road surface using special algorithms for controlling the longitudinal slip of a tyre. A research vehicle with electromechanical brake callipers controlled by brake-by-wire technology was used here. To conclude, the authors of the article [10], on the basis of their experimental results, state that electromechanical brake actuators and brake-by-wire solutions allow for the high performance and robustness of the anti-lock brake system.

The work [11] presents a method for controlling the fuzzy distribution of drive torque between individual wheels driven by electric motors. The engine control is dependent on the adhesion conditions between a tyre and a road surface. The authors of the work [11] conclude that their presented algorithm allowed for the improvement in driving stability and fuel economy of the in-wheel motor vehicle, under complex driving conditions.

The paper [12] presents research aimed at improving the operation of an ABS system, using CarSim programming, by maximizing longitudinal adhesion coefficient of a tyre to a road surface.

The article [13] presents a method for estimating the adhesion coefficient of a tyre to a road surface, using a simple bicycle model and one of the image analysis methods.

The article [14] presents an improved model of ABS (anti-lock braking system). In particular, a control system that takes into account various wheel load conditions resulting from a vehicle manoeuvre is presented here. The evaluation of the performance of the ABS under various operating conditions is shown in the article [14].

Most of the studies discussed here used advanced measurement equipment to determine the forces acting on the wheels and their slip, which often required intervention in the structure of a vehicle. A method similar to the one discussed in this study can be found in a doctoral thesis [15], where the acquisition of a vehicle’s longitudinal acceleration was used to determine a tyre-to-road adhesion coefficient (in this case called road grip).

The article [16] presents the influence of the changes in the properties of the braking system of a tractor–semitrailer set on the braking process, including the risk of jack-knifing phenomena.

In the paper [17], there is a discussion of the problem of longitudinal and lateral dynamics of vehicle motion, including a correlative–optical sensor of vehicle velocity. The authors of this work included unscented Kalman filtration and estimation of side slip angles, taking into account tyre-to-road friction changes.

In the article [18], it is shown that the model that describes the influence of the temperature on tyre-to-road friction coefficient, including rotational velocity of a wheel and the size of the area of the contact between the wheel and the road surface.

The work [19] discusses different devices designed for the measurement of skid resistance. The authors also mention the pavement skid resistance according to both longitudinal and lateral direction.

The work [4], co-authored by some of the co-authors of this article, shows the skid resistance tester SRT-4 that is widely applied by the laboratories of General Directorate for National Roads and Motorways in Poland. The system is designed for investigating the anti-skid properties of the road surfaces, which is the part of interest for civil engineering. The SRT-4 system is also widely used by the authors of the paper [4] for the needs of automotive engineering, especially to find the tyre-to-road adhesion characteristics in different weather conditions and different tyre types. Such approach is limited to the braked wheel issue, which is definitely a different issue compared to the driven wheel.

The authors of the article [20] discuss tyre-to-road friction interaction in dependence with the unevenness of the road and wet road surface conditions. To do this, the authors implemented a three-dimensional model of uneven road and, to simulate wet road surface properties, the pseudo-hydrodynamic bearing effect between tyre and road surface was applied.

In the article [21] is discussed the application of the finite element method in modelling tyre deflection dynamics and its influence on tyre-to-road friction coefficient.

In the work [22] is presented the way the tyre-to-road peak friction coefficient is obtained with the use of the vehicle dynamics model and extended Kalman filter.

The authors of the work [23] show that the model of a tyre presented in their article allowed for identifying the instant adhesion properties between tyre and road and estimation of the dissipated power.

In the article [24], the authors present the review of estimation techniques of road friction including slip-based approaches as well as low and high-frequency vibration-based methods.

In the article [25] is shown the strategy of controlling the wheels’ slip during accelerating in case of four-wheel drive vehicle with the variant of constant front and rear wheels torque transmission.

The work [26] shows the approach to automotive wheel modelling with the use of finite element analysis, including the Mooney–Rivlin material model implemented to describe the hyper-elastic properties of the tyre rubber elements.

The article [27] shows the application of signals from the OBD2 system of automobiles using the ELM 327 interface in predicting the vehicle performance curves and acceleration responses. The usefulness of the artificial neural networks (ANNs) is also shown in the article [27], as well as the applied quarter-car suspension model of vertical vibrations and the approach to the transient conditions of a powertrain. The common feature of the article authored by Sobrino et al. is that our work is also based on the signals acquired from the OBD2/EOBD (DLC-3—Data Link Connector 3) automotive data interchange interface. Such approach does not exclude the application of separate external sensors, but it is cheaper as the sensors are already present on the board of a vehicle.

The work [28] presents the real-time tyre–road friction coefficient methodology based on the estimation of friction coefficient and the detection of abrupt changes in its value. The work [28] shows that it is possible to estimate the friction coefficient on the basis of vehicle longitudinal motion measurements with appropriate vehicle longitudinal dynamics and puts the pressure on the explanation of a few different models of a tyre often applied in many works.

The work [29] discusses the problem of the measurement of adhesion force between a tyre and a road surface, through the CAD (computer-aided design) model and in support of the MSC ADAMS software.

Accelerated motion of an automobile is investigated not only because of the adhesion phenomenon between a tyre and a road surface. It is also crucial because of the exhaust emission, as depicted in the work [30], wherein the authors differentiate between different vehicle acceleration styles.

Another paper in which vehicle advanced model is applied, is the work [31]. The authors applied a model which was prepared to include the vibrations and the stability of vehicle motion, including twelve degrees of freedom.

In Section 3 of this article will be presented the methodology to obtain the dependence between tyre-to-road surface longitudinal adhesion coefficient and wheel longitudinal slip ratio (often called adhesion characteristic) in driven wheel conditions. Such approach is not popular in scientific articles when discussing driven wheels. As mentioned above, based on the literature reviewed, it can be stated that the tyre–road adhesion is discussed much more often in the case of a braked wheel than in the case of a driven wheel. Therefore, it is justified to conduct research in the less popular field of driven wheel adhesion, simultaneously applying the on-board data of automobiles. The novelty of the presented methodology is the possibility of obtaining the mentioned characteristic on the basis of a single acceleration attempt, with the application of relatively simple and cheap measurement equipment, partly based on the sensors present on the board of the automobile.

## 3. Method for Determining Adhesion Characteristics During Acceleration

**Figure 2:**
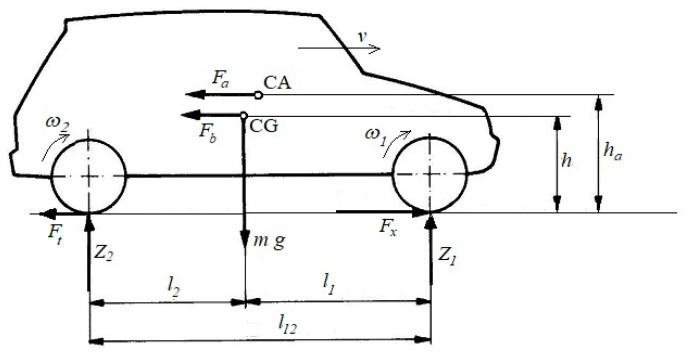
The system of the forces acting on a vehicle during acceleration.

Figure 2 shows the system of the forces acting on a vehicle during acceleration.

The following symbols are used in Figure 2 and in the formulas presented later in the article (additional symbols together with their values are depicted in Table 1):

*v*—vehicle velocity;
*a*—acceleration of a vehicle;
*CG*—centre of mass;
*CA*—centre of air pressure;
*F_x_*—longitudinal adhesion force of the front wheels;
*Z*_1_, *Z*_2_—normal reactions acting on the wheels of the front and rear axles;
*F_b_*—inertial resistance force;
*F_a_*—air drag force;
*F_t_*_2_—rolling resistance force of the rear wheels;
*ω*_1_, *ω*_2_—angular velocities of the front and rear wheels (averaged for the left and right wheels);
*n*_1_, *n*_2_—rotational velocities of the front and rear wheels (averaged for the left and right wheels);
*r_t_*_1_, *r_t_*_2_—rolling radius of the front and rear wheels;
*r_d_*_1_, *r_d_*_2_—dynamic radius of the front and rear wheels;
*f_t_*—rolling resistance coefficient;
*S*_1_, *S*_2_—slip of front and rear wheel.

In Table 1, the values of the parameters of the automobile applied in presented analysis are depicted.

The balance of the forces acting on an accelerated front-wheel driven automobile (Figure 2) shows that the adhesion force developed by the front wheels *F_x_* must balance the motion resistance forces: the rolling resistance force of the rear wheels *F_t_*_2_, the inertial resistance force *F_b_* and the air resistance force *F_a_*. This gives Equation (4) of the motion for a vehicle.

(4) $$F_{x}=F_{t2}+F_{b}+F_{a},$$

The formula does not take into account the rolling resistance force of the front wheels because the adhesion force of these wheels is not used to overcome it. The rolling resistance force of the rear wheels depends on the normal force *Z*_2_ and the rolling resistance coefficient *f_t_*, which in turn depends on a type of a surface and increases with the increasing velocity of a vehicle. A quadratic relationship is usually assumed here.

(5) $$F_{t2}=Z_{2}{\cdot \;f}_{t},$$

(6) $$f_{t}=0.01\cdot \left(1+k_{t}\;\cdot v^{2}\right),$$

Inertial resistance force:

(7) $$F_{b}=m\;\cdot a,$$

Air drag force:

(8) $$F_{a}=0.047\cdot \;A\;\cdot c_{x}\cdot \;v^{2},$$

where

- *v*—velocity of automobile expressed in km/h (wind velocity is assumed as equal to zero).

Longitudinal forces (inertial resistance force and air drag force) acting on the body of a vehicle cause changes in the vertical axle loads. Therefore, the normal reaction acting on the wheels of front axle is as follows:

(9) $$Z_{1}=\frac{m\cdot \;g\;\cdot l_{2}-F_{b}\;\cdot h-F_{a}\;\cdot h_{a}}{l_{12}},$$

and normal reaction force acting on the wheels of the rear axle

(10) $$Z_{2}=\frac{m\;\cdot g\;\cdot l_{1}+F_{b}\;\cdot h+F_{a}\;\cdot h_{a}}{l_{12}},$$

Therefore, the increase in the load (practically decrease) of the front wheels:

(11) $${\Delta Z}_{1}=\frac{-F_{b}\;\cdot h-F_{a}\cdot \;h_{a}}{l_{12}},$$

and the increase in the rear wheels’ load:

(12) $${\Delta Z}_{2}=\frac{F_{b}\;\cdot h+F_{a}\cdot \;h_{a}}{l_{12}},$$

The dynamic radius of the wheel (distance of the wheel axle from the road surface) increases with the increase in vehicle velocity (with the proportionality coefficient *k_v_* assumed here) and decreases as a result of the wheel load. The normal load of the front wheels is decreasing when accelerating (negative increase in normal load).

(13) $$r_{d1}=r_{stat}+k_{v}\cdot v-{∆Z}_{1}/k_{r}\;,$$

And in case of the front wheels, there is positive increase in normal load

(14) $$r_{d2}=r_{stat}+k_{v}\cdot v-{∆Z}_{2}/k_{r}\;,$$

The above presented Formulas (13) and (14) are presented in this article only for discussion aims and show one of the possibilities to estimate the dynamic radius of a wheel. So the values of the coefficients such as *k_v_*, *r_stat_* are not presented in this article.

In a front-wheel driven automobile, the front wheels’ slip occurs when accelerating. However, it can be assumed that the rear wheels roll without slip and their circumferential velocity *ω*_2_*·r_d_*_2_ is equal to a vehicle velocity *v*. The slip of the front wheel can be deter-mined from the following relationship:

(15) $$S_{1}=\frac{\omega_{1}{\cdot r}_{d1}-v}{\omega_{1}\cdot r_{d1}},$$

Replacing the velocity *v* by circumferential velocity of the rear wheel *ω*_2_*·r_d_*_2_ gives

(16) $$S_{1}=\frac{\omega_{1}\cdot r_{d1}-\omega_{2}\cdot r_{d2}}{\omega_{1}\cdot r_{d1}},$$

The angular velocities of the front and rear wheels can be calculated from the measured rotational velocities *n*_1,2_:

(17) $$\omega_{1,2}=\frac{2\cdot \;\pi \;\cdot n_{1,2}}{60},$$

where

1, 2—front and rear wheels, respectively.

In case that it was a straight-line motion, for the calculations the arithmetic mean of the left and right wheel angular velocity was used. So, *n*_1_ and *n*_2_ are the rotational velocities of, respectively, the front and rear axle, calculated as the arithmetic mean of the rotational velocity of the left and right wheel.

As it was already commented according to Equations (13) and (14), the alternative formulas for the estimation of dynamic radius are presented as the relationship between the dynamic radius of the front and rear wheels. The values of the dynamic radius can be determined using Formulas (18) and (19):

(18) $$r_{d1}=r_{d2}+\left({∆Z}_{2}-{∆Z}_{1}\right)/k_{r},$$

Assuming that there is no slip of the rear wheels, the dynamic radius of rear wheels can be calculated as follows:

(19) $$r_{d2}=\frac{v}{\omega_{2}},$$

Using Formula (15) and the relationships from the Formulas (17)–(19), it is possible to determine the slip of the front wheels *S*_1_ based on the measured rotational velocities of the front wheels *n*_1_ and the rear wheels *n*_2_ and the vehicle velocity *v*.

The adhesion force of the front wheels *X*_1_ is determined based on Formula (4) as equal to the driving force *F_x_* developed by these wheels and the value of the normal reaction acting on these wheels *Z*_1_ is determined on the basis of Formula (9) to allow for the determination of the adhesion coefficient of these wheels according to Formula (1).

(20) $$\mu_{x}=\frac{F_{x}}{Z_{1}},$$

where

- *F_x_*—driving force developed by front wheels;
- *Z*_1_—normal reaction acting on front wheels.

## 4. Road Tests

**Figure 3:**
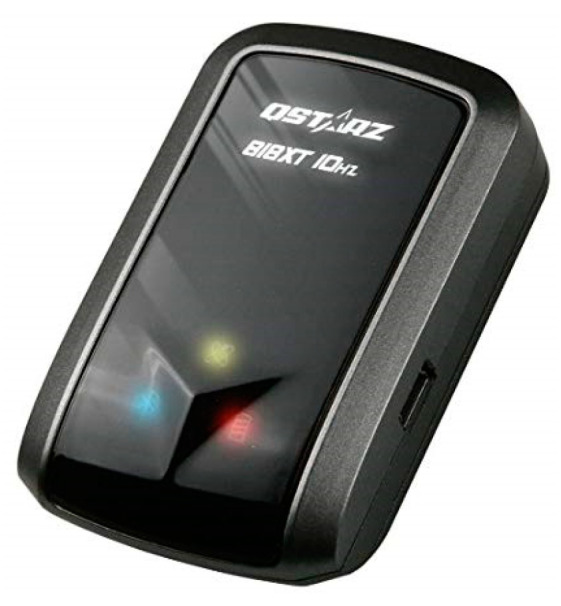
Qstarz BT818XT 10 Hz GPS receiver applied in the research [32].

**Figure 4:**
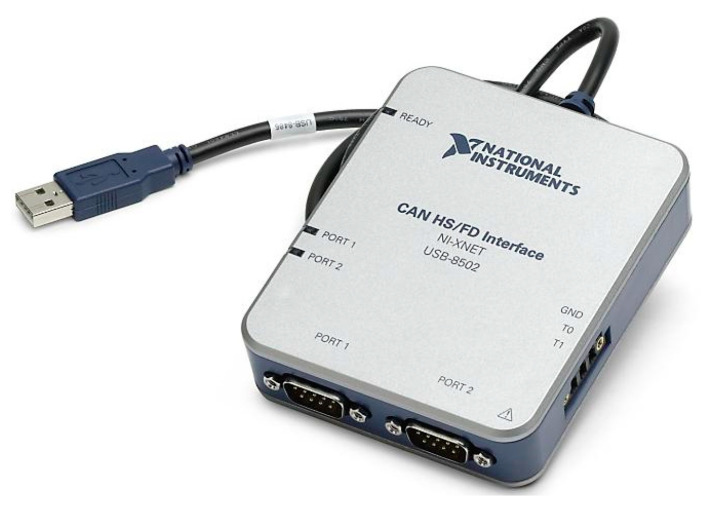
National Instruments measurement card NI-XNET USB-8502 applied in the research [33].

The object of this research was middle-class passenger automobiles with front-wheel drive. The road surface was dry, clean asphalt. The vehicle’s anti-skid system (ASR/TCS) was deactivated. The road measurements carried out were aimed at recording the vehicle velocity and recording the rotational velocities of driven and non-driven wheels. The vehicle velocity signal was obtained from the GPS receiver [32] (see Figure 3), and the wheels’ rotational velocity signals were obtained from the vehicle CAN bus via a special measurement card [33] (see Figure 4).

**Figure 5:**
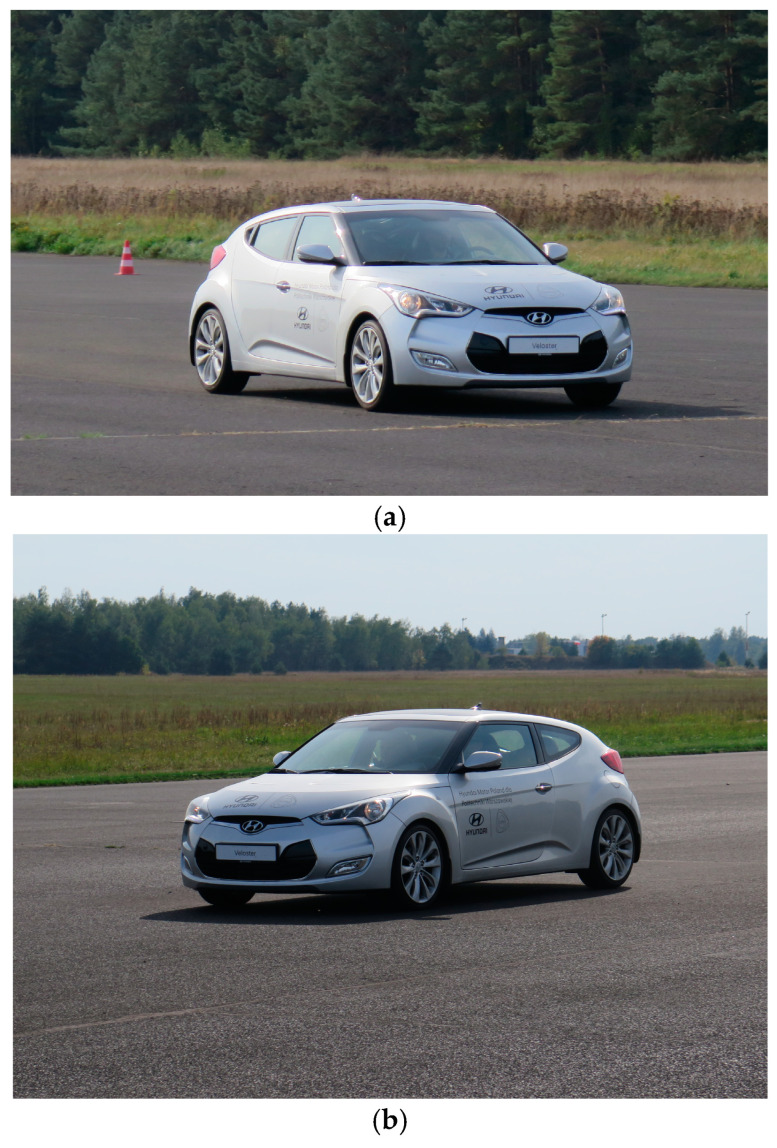
Measurement vehicle (Hyundai Veloster) on the research venue (photos of the authors): (**a**) a photo from the right side, (**b**) a photo from the left side.

Figure 5 shows the measurement vehicle during the tests conducted on the research venue.

Thanks to a special program developed in the LabVIEW environment, it was possible to record the wheels’ velocity signals from the automotive CAN network and the vehicle velocity from the GPS receiver and to synchronize these signals.

**Figure 6:**
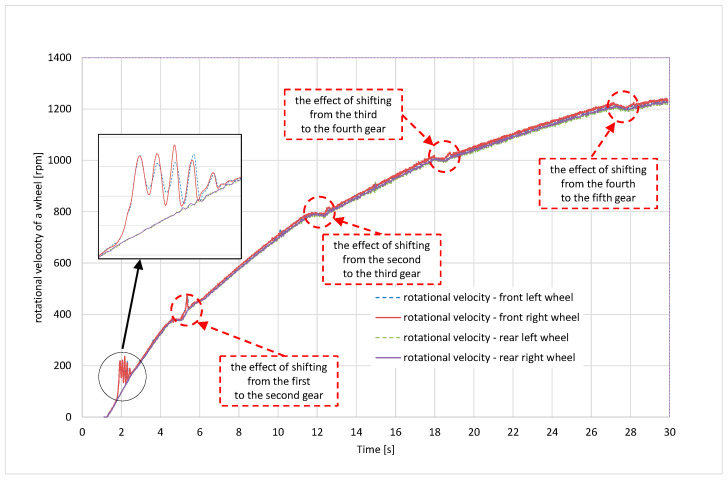
Rotational velocities of the front and rear wheels when accelerating.

Figure 6 shows the rotational velocities of the wheels in time domain, acquired from the CAN bus of the automobile through the above-mentioned CAN bus card and LabView program. Additionally, in Figure 6 are depicted the ranges in which the gears were changed. The enlarged fragment of the graph for the first gear shows increased values of the wheels’ rotational velocities, which may result from self-excited wheels’ vibrations caused by momentary loss of tyre adhesion. 

In order to obtain the highest possible value of driving force, which would result in a high value of slip of the driven wheels, the vehicle was accelerated with the highest possible acceleration, but not so high as to result in loss of adhesion.

**Figure 7:**
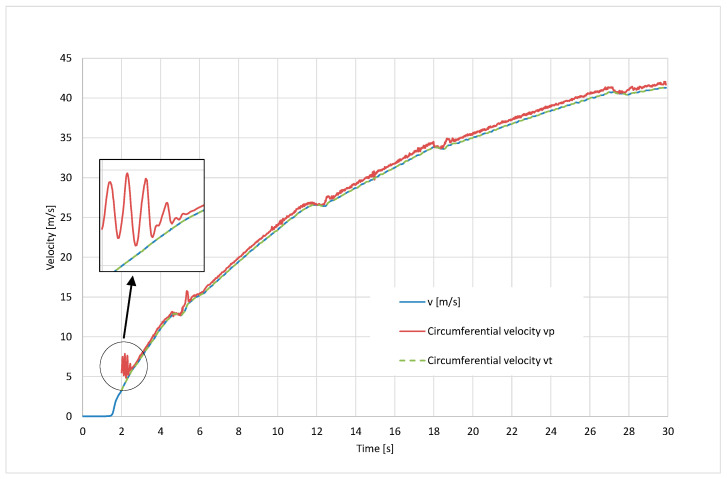
Vehicle velocity and circumferential velocities of the front and rear wheels when accelerating.

Figure 7 shows the time course of the vehicle velocity recorded based on the signal from the GPS receiver and the average circumferential velocities of the front wheels *v_p_* and rear wheels *v_t_*, determined on the basis of rotational velocities (from the CAN bus) taking into account the changes in dynamic radii according to Formulas (13) and (14).

The graph shows that the circumferential velocity of the front (driven) wheels is higher than that of the rear wheels, which indicates the slip of the driven wheels. The circumferential velocity of the undriven rear wheels coincides with the vehicle velocity, which means that the circumferential velocity of the rear wheels can be used as the vehicle velocity signal. In the initial phase of the acceleration process, as well as after engaging the second gear, visible pulsations of the circumferential velocity of the front wheels can be observed, which can be interpreted as their self-excited vibrations caused by a momentary loss of the adhesion when the driving force is too high. These parts of the graph will not be taken into account in the further part of the calculations.

**Figure 8:**
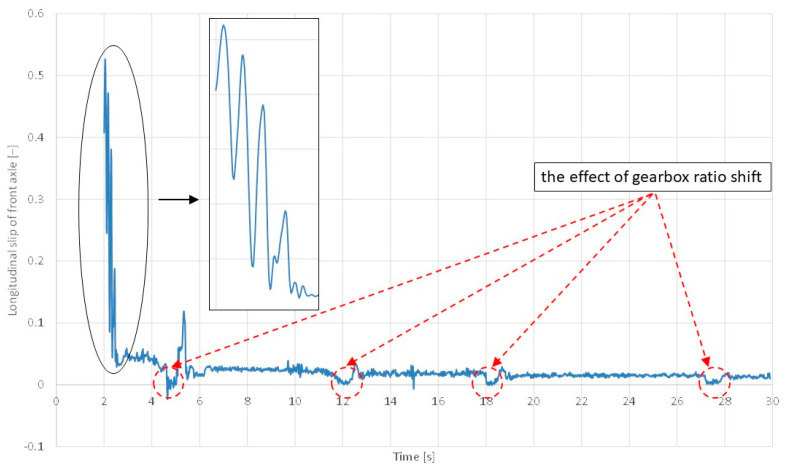
Front wheels slip in time domain.

Based on the difference in the circumferential velocity of the front wheels and the vehicle velocity, the slip of the front wheels was determined (see Figure 8). As can be seen in the course of the estimated longitudinal slip ratio of front axle depicted in Figure 8, the harmonic changes in the slip can be clearly seen for the first gear. This may be related to self-excited vibrations on the patch between the tyre and the road surface. On the other gears (from the second to the fifth gear), we see very low values of the longitudinal slip ratio, which are decreasing to zero nearing the change into the following gear (approximately 5, 12, 18, 27.5 s). Simultaneously, during the gearbox ratio shift from first to the second gear, nearing 5 s appeared the negative values of the longitudinal slip of the front axle in the range of time approximately 0.2 s wide. This may be due to the fact that, when the driving force suddenly disappears during a gear change, the drop in the circumferential velocities of the wheels is higher than the drop in the forward velocity of the vehicle, resulting in negative slippage (as when braking). There is another case (at a time instant of approximately 15 s), wherein longitudinal slip also decreases below zero. In this case, the effect determined by the unevenness of the road surfaces may occur, thereby causing vertical vibrations of the unsprung masses of the automobile and disturbing the signal of angular velocity of the wheels. Self-excited wheels’ vibrations can be seen in Figure 8.

**Figure 9:**
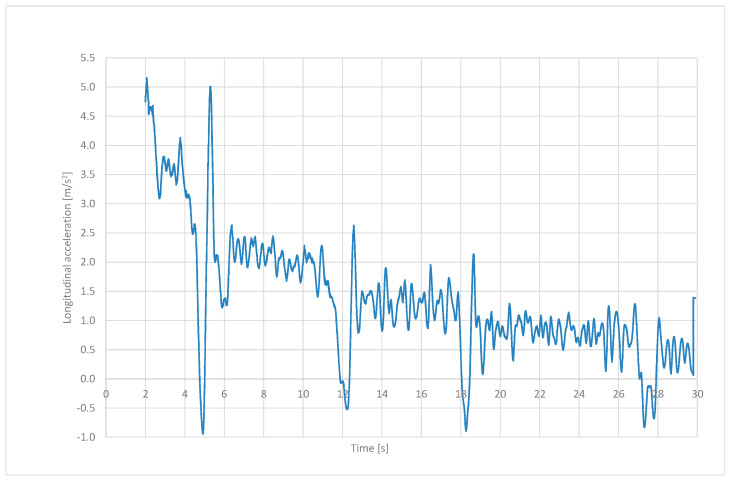
Longitudinal acceleration in time domain.

Accelerations were determined based on the velocity versus time graph (Figure 9). Characteristic sudden changes in the longitudinal acceleration values of the vehicle can be observed during the gear shifting.

## 5. Discussion

**Figure 10:**
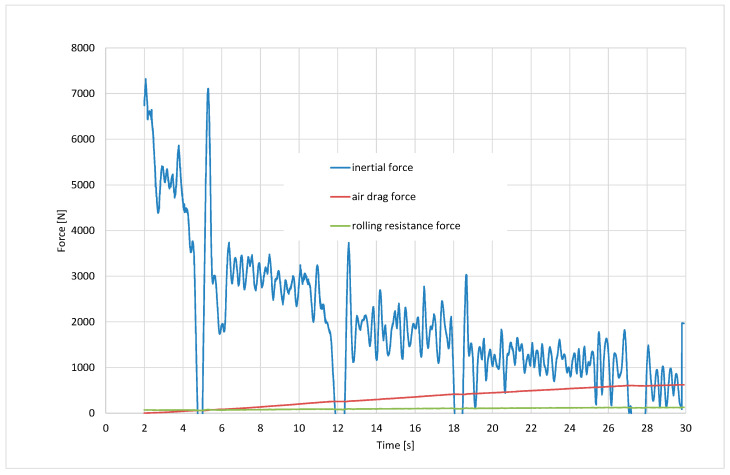
Drag forces of the vehicle motion.

Calculating the adhesion coefficient according to Formula (20) as the ratio of the tangential force to the normal force required the prior calculation of the vertical reaction of the road loading the front wheels *Z*_1_ and the driving force *F_x_* transmitted by these wheels. When determining the vertical reaction of the front wheels, their unloading resulting from the inertial resistance force and air drag force was taken into account, according to Formula (11). When determining the driving force transmitted by the front wheels, it was assumed that it balances the sum of the motion resistance forces according to Formula (4), which consists of the rolling resistance force of the rear wheels, the air drag force, and the inertial resistance force (Figure 10).

**Figure 11:**
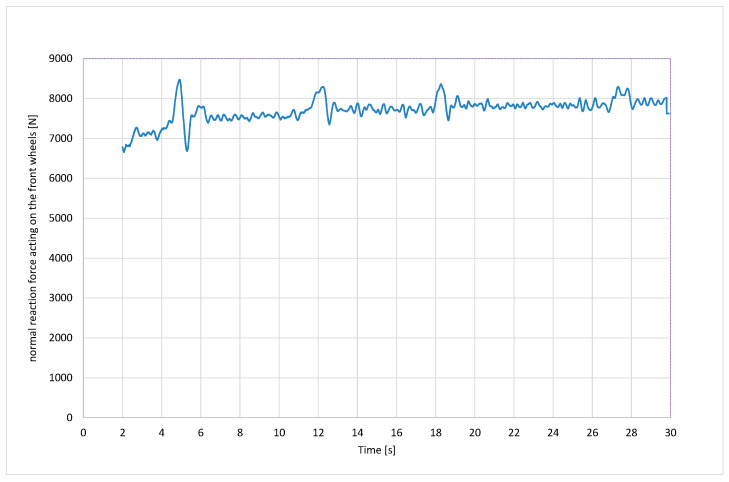
Normal reaction force acting of the front wheels.

Figure 11 shows the normal reaction force of the front wheels calculated from Equation (9). The value of the longitudinal adhesion coefficient can be obtained by dividing the sum of the resistance forces from the graph in Figure 10 by the corresponding value of the normal reaction force from the graph in Figure 11.

**Figure 12:**
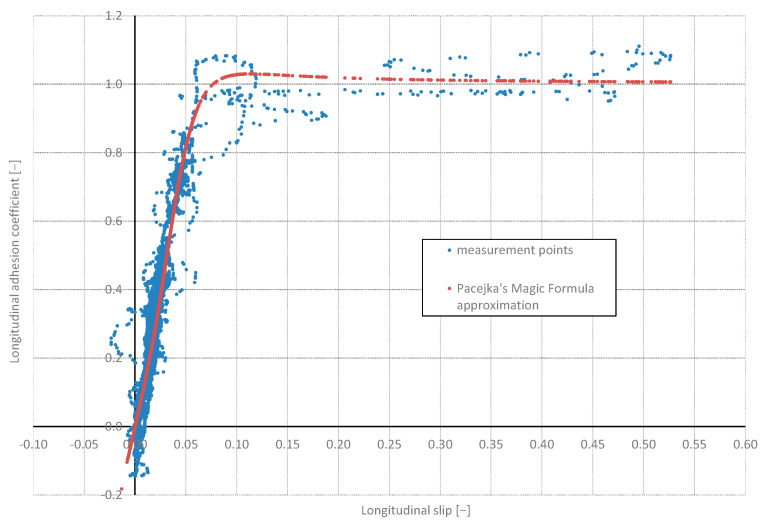
Adhesion characteristics (after removing the points from the beginning of the test with the highest pulsations in the rotational velocity of front wheels).

After determining the slip value according to Formula (15) and the values of adhesion coefficients according to Formula (1), it is possible to prepare a graph of adhesion characteristics (adhesion coefficient as a function of slip, Figure 12) for the selected tyre–road surface pair based on the measured wheel rotational velocities and velocity of the automobile. For comparison, a Magic Formula curve corresponding to adhesion according to Pacejka’s Formula (21) [34] for a wheel loaded with longitudinal force is plotted on the same graph.

(21) $$\mu_{x}=D\cdot sin\left(C\cdot atan\left(B\cdot S_{1}-E\cdot \left(B\cdot S_{1}-atan\left(B\cdot S_{1}\right)\right)\right)\right),$$

The Magic Formula approximation curve was prepared for the following coefficients: *B* = 11, *C* = 1.15, *D* = 1.03, *E* = −10. The coefficients of the Magic Formula were used here as examples. To apply the Magic Formula approximation for the simulation properties, it should be preceded by the identification of the following coefficients of Formula (21). The presented approximation method is widely applied for the analytical description of the dependence of longitudinal adhesion coefficients and longitudinal slip ratio. It is also applied for the dependence between lateral force acting between a wheel and a side slip angle. The main reason is that the curve resulting from Pacejka’s Equation (21) is “flexible” enough to match the desired course of the dependences discussed above.

## 6. Conclusions

The presented method confirmed that it is possible to obtain the adhesion characteristic as tyre-to-road longitudinal adhesion coefficient in wheel longitudinal slip domain, for the case of the driven wheel on the basis of a single acceleration attempt. The presented work is original as it introduces the experiment of the driven wheel approach, which is not popular contrary to the braked wheel, which is widely investigated in practice (for example, with the application of the SRT-4 system, as mentioned in Section 2). The presented method is relatively simple and cheap as it needs relatively cheap measurement equipment. The method should be verified in a way that is described below in this chapter.

The performed tests confirmed the possibility of determining the graph of adhesion coefficient as a function of slip, or more precisely, a fragment of this graph from the zero value to points close to the peak adhesion coefficient (see Figure 12). In the future, the authors of this article are planning to develop the following methodology:

1. The comparison of the signals recorded from the CAN bus with the signals acquired from additionally mounted sensors that would duplicate the signals from the CAN bus, the application of a correlative–optical velocity sensor mounted on the automobile instead of a simple GPS receiver, improvement of the measured signals management through the Butterworth lowpass filtering procedure;
2. Vibrations occurring near the maximum of the graph, resulting from entering the unstable range of the adhesion characteristics, make it practically impossible to precisely determine the value of the adhesion and slip coefficient corresponding to this value—the filtering procedure would improve it;
3. The practical use of the proposed method in diagnosing the condition of the road surface will therefore depend on solving the above-mentioned difficulties and the possibility of conducting calculations in real time;
4. The experiments should be repeated also for wet, snowy, and icy road surfaces, which would allow for the assessment of the sensitivity of the proposed methodology;
5. As revealed in Figure 5, during dynamic acceleration on the first gear, there may be self-excited vibrations between the tyre and the road surface, which may be in the future the basis of further investigations, including a model of friction; such phenomenon may have an influence on the durability of powertrain elements (a slip on a clutch between an engine flywheel and an input shaft of a gearbox, torsional vibrations of a crankshaft of an internal combustion engine);
6. As can be seen from Figure 6, Figure 7, Figure 8 and Figure 9, the moment the gears shift is clearly visible—it would be interesting to repeat the investigation for automobiles equipped with dual-clutch transmission and to compare the results with the ones performed for the manual gearbox.

## Figures and Tables

**Table 1:** Parameters of automobile applied in the analysis.

Parameter of Automobile	Value of a Parameter
mass of a vehicle *m* [kg]	1419
front cross-sectional area of a vehicle *A* [m^2^]	2
height of the centre of mass *h* [m]	0.56
height of the centre of air pressure *h_a_*	0.7
wheelbase *l*_12_	2.65
distance of the centre of mass from the front and rear axles *l*_1_, *l*_2_ [m]	1.089, 1.561
radial stiffness of a tyre *k_r_* [N/m]	500,000
coefficient of the influence of a vehicle velocity on the rolling resistance coefficient *k_t_* [s^2^/m^2^]	5·10^−5^
air drag coefficient of automobile *c_x_* [-]	0.30

## Data Availability

Data are contained within the article.

## References

[1] Diagnostyka stanu nawierzchni i wybranych elementów korpusu drogi Wytyczne Stosowania. Warsaw, May 2019. Generalna Dyrekcja Dróg Krajowych i Autostrad. https://www.archiwum.gddkia.gov.pl/pl/2982/Diagnostyka-Stanu-Nawierzchni.

[2] Regulation No 117 of the Economic Commission for Europe of the United Nations (UNECE)—Uniform Provisions Concerning the Approval of Tyres with Regard to Rolling Sound Emissions and/or to Adhesion on Wet Surfaces and/or to Rolling Resistance [2016/1350]. https://eur-lex.europa.eu/legal-content/EN/TXT/PDF/?uri=CELEX:42016X0812(01).

[3] Mechowski T. (2009). Measurement of the Road Friction Coefficient in Poland. Proc. Inst. Veh..

[4] Pokorski J., Reński A., Sar H. (2015). System for Investigation of Friction Properties of the Road Surface. Balt. J. Road Bridge Eng..

[5] Sanders P.D., Browne C. (2020). Characterising the Measurements Made by Sideways-Force Skid Resistance Devices. A Desk Study and Proposal for an Experimental Study. TRL. The Future of Transport. Published Project Report PPR957. https://trl.co.uk/uploads/trl/documents/PPR957---Characterising-the-measurements-made-by-sideways-force-skid-resistance-devices.pdf.

[6] Salehi M., Noordermeer J.W.M., Reuvekamp L.A.E.M., Tolpekina T., Blume A. (2020). A New Horizon for Evaluating Tire Grip Within a Laboratory Environment. Tribol. Lett..

[7] Cabrera J.A., Castillo J.J., Pérez J., Velasco J.M., Guerra A.J., Hernández P. (2018). A Procedure for Determining Tire-Road Friction Characteristics Using a Modification of the Magic Formula Based on Experimental Results. Sensors.

[8] Singh K.B., Taheri S. (2015). Estimation of tire–road friction coefficient and its application in chassis control systems. Syst. Sci. Control. Eng..

[9] Villagra J., d’Andrea-Novel B., Fliess M., Mounier H. An algebraic approach for maximum friction estimation. Proceedings of the 8th IFAC Symposium on Nonlinear Control Systems (NOLCOS).

[10] Petersen I., Johansen T.A., Kalkkuhl J., Lüdemann J. Wheel slip control using gain-scheduled LQ—LPV/LMI analysis and experimental results. Proceedings of the 2003 European Control Conference (ECC), 2003. Presented at the 2003 European Control Conference (ECC).

[11] Park J., Jeong H., Jang I.G., Hwang S.-H. (2015). Torque Distribution Algorithm for an Independently Driven Electric Vehicle Using a Fuzzy Control Method. Energies.

[12] Hoseinnezhad R., Bab-Hadiashar A. (2011). Efficient Antilock Braking by Direct Maximization of Tire-Road Frictions. IEEE Trans. Ind. Electron..

[13] Tian C., Leng B., Hou X., Xiong L., Huang C. (2022). Multi-Sensor Fusion Based Estimation of Tire-Road Peak Adhesion Coefficient Considering Model Uncertainty. Remote. Sens..

[14] Bera T.K., Bhattacharya K., Samantaray A.K. (2011). Evaluation of antilock braking system with an integrated model of full vehicle system dynamics. Simul. Model. Pract. Theory.

[15] Pinto Braz J.P. (2019). A Novel Road Grip Estimation Method Using a Vehicle as a Probe. Ph.D. Dissertation.

[16] Radzajewski P., Guzek M. (2023). Assessment of the Impact of Selected Parameters of Tractor-Semitrailer Set on the Braking Safety Indicators. Appl. Sci..

[17] Alshawi A., De Pinto S., Stano P., van Aalst S., Praet K., Boulay E., Ivone D., Gruber P., Sorniotti A. (2024). An Adaptive Unscented Kalman Filter for the Estimation of the Vehicle Velocity Components, Slip Angles, and Slip Ratios in Extreme Driving Manoeuvres. Sensors.

[18] Cattani P., Cattani L., Magrini A. (2023). Tyre–Road Heat Transfer Coefficient Equation Proposal. Appl. Sci..

[19] Rosta S., Gáspár L. (2023). Skid Resistance of Asphalt Pavements. Eng.

[20] Zhang L., Wang R., Zhou H., Wang G. (2022). Estimation of the Friction Behaviour of Rubber on Wet Rough Road, and Its Application to Tyre Wet Skid Resistance, Using Numerical Simulation. Symmetry.

[21] Maknickas A., Ardatov O., Bogdevičius M., Kačianauskas R. (2022). Modelling the Interaction between a Laterally Deflected Car Tyre and a Road Surface. Appl. Sci..

[22] Han Y., Lu Y., Chen N., Wang H. (2022). Research on the Identification of Tyre-Road Peak Friction Coefficient under Full Slip Rate Range Based on Normalized Tyre Model. Actuators.

[23] Roveri N., Pepe G., Mezzani F., Carcaterra A., Culla A., Milana S. (2019). OPTYRE—Real Time Estimation of Rolling Resistance for Intelligent Tyres. Sensors.

[24] Acosta M., Kanarachos S., Blundell M. (2017). Road Friction Virtual Sensing: A Review of Estimation Techniques with Emphasis on Low Excitation Approaches. Appl. Sci..

[25] He H., Peng J., Xiong R., Fan H. (2014). An Acceleration Slip Regulation Strategy for Four-Wheel Drive Electric Vehicles Based on Sliding Mode Control. Energies.

[26] Fathi H., El-Sayegh Z., Ren J., El-Gindy M. (2024). Modeling and Validation of a Passenger Car Tire Using Finite Element Analysis. Vehicles.

[27] Sobrino y Arjona-Guzman M., Jimenez-Martinez M., Torres-Cedillo S.G. (2022). Vehicle Performance Assessment Using the OBD2 Port and Artificial Neural Network. Eng. Lett..

[28] Rajamani R. (2012). Tire-Road Friction Measurement on Highway Vehicles. Vehicle Dynamics and Control. Mechanical Engineering Series.

[29] Jilek P. (2023). Vehicle wheel positioning innovation on a machine for measuring the contact parameters between a tyre and the road. Arch. Automot. Eng. Arch. Motoryz..

[30] Pečman J., Šarkan B., Ližbetinová L., Ľupták V., Loman M., Bartuška L. (2024). Impact of Acceleration Style on Vehicle Emissions and Perspectives for Improvement through Transportation Engineering Solutions. Arch. Automot. Eng. Arch. Motoryz..

[31] Akhmedov D., Riskaliev D. (2024). Modeling of full vehicle dynamics for enhanced stability control. Arch. Automot. Eng. Arch. Motoryz..

[32] GPS Receiver BT-Q818XT Extreme 10 Hz. http://racing.qstarz.com/Products/BT-Q818XT.html.

[33] National Instruments Measurement Card USB-8502. https://www.ni.com/pl-pl/shop/model/usb-8502.html.

[34] Pacejka H.B. (2012). Tire and Vehicle Dynamics.

